# Pregabalin for Opioid-Refractory Pain in a Patient with Ankylosing Spondylitis

**DOI:** 10.1155/2013/912409

**Published:** 2013-06-17

**Authors:** Konstantinos A. Kontoangelos, Anastasios V. Kouzoupis, Panagiotis P. Ferentinos, Ioannis D. Xynos, Nikolaos V. Sipsas, George N. Papadimitriou

**Affiliations:** ^1^1st Department of Psychiatry, Eginition Hospital, Athens University Medical School, 74 Vas Sofias Avenue, 11528 Athens, Greece; ^2^University Mental Health Research Institute, 11527 Athens, Greece; ^3^Pathophysiology Department, Laikon General Hospital, Athens University Medical School, 11528 Athens, Greece

## Abstract

*Background*. Ankylosing spondylitis (AS) is a systemic inflammatory disease with chronic back pain as the most common presenting symptom. We present a case of a male patient with AS reporting symptoms of severe low back pain, buttock pain, and limited spinal mobility. After chronic treatment with opioids, we administered pregabalin at a dose of 300 mg as an analgesic agent while opioids were discontinued. *Findings*. Pain symptoms improved progressively, and opioids were gradually discontinued without any withdrawal symptoms reported. *Conclusions*. Pregabalin is potentially useful in the management of pain in patients with AS while effectively managing the discontinuation of opioid treatment.

## 1. Introduction

Ankylosing spondylitis (AS) is a systemic inflammatory disease of unknown origin that affects predominantly, but not exclusively, male individuals. Chronic inflammatory back pain is the most common presenting symptom and typically develops between the ages of 20 and 40. Alternating buttock pain caused by sacroiliitis is a less frequent presenting symptom but is often regarded as a classic initial indication of the disease [1].

Management of AS is based on both nonpharmacological and pharmacological treatment options [2]. Nonpharmacological treatments include education, exercise, physiotherapy, and surgical interventions. Pharmacological agents used in the treatment of AS involve nonsteroidal anti-inflammatory drugs (NSAIDs, conventional ones and coxibs), disease modifying antirheumatic drugs such as sulphasalazine and methotrexate, biological agents such as etanercept and infliximab, steroids (local and systemic), and analgesics. Opioid analgesics, in particular, might be considered for pain control in AS patients in whom NSAIDS are inefficient, contraindicated, and/or poorly tolerated [3]. A number of anticonvulsants are effective for chronic pain therapy, particularly for lancinating pain [4]. Phenytoin, carbamazepine, oxcarbazepine, valproic acid, and clonazepam, as well as the newer agents, gabapentin and pregabalin, have been used frequently with impressive results. Pregabalin, like gabapentin, was shown to be effective in several forms of neuropathic pain, incisional injury, inflammatory injury, and formalin-induced injury. [5–7].

We present a case in which pregabalin was reported effective in a male patient with AS suffering from opioid-refractory pain.

## 2. Case Presentation 

Mr. A. was a 72-year-old male patient with AS, reporting symptoms of low back pain, buttock pain, shoulder pain, arthralgias, and limited spinal mobility. The intensity of the pain that the patient experienced was described from him as a severe stabbing and shooting pain, splitting and exhausting, sickening, and very fearful causing him severe discomfort. He had been suffering from AS since the age of 47. On admission in February 2008, the patient reported that during the previous two years he had been prescribed a combination of various medications for pain relief with only transient improvement. When admitted to the hospital, he was on the following treatment for the last six months: fentanyl 400 mcg sbl once a day, paracetamol 500 mg twice a day, and codeine 30 mg p.o. twice a day. On the Short-Form McGill Pain Questionnaire [8], he scored I-a = 24, II = worst possible pain, III = 2, while on the Beck Depression Inventory (BDI) [9] he had a score of 24. His score on BDI was mainly shaped from his reported symptoms in the subscale that measures somatic-vegetative performance complaints (consisting of the last eight items of the BDI). Pregabalin was gradually administered, with a starting dose of 75 mg once a day that over a period of two weeks increased to 75 mg four times a day. Pregabalin is a new agent that exerts its pharmacodynamic effect by modulating voltage-gated calcium channels and has a linear pharmacokinetic profile. It is completely absorbed, not bound to plasma proteins, not metabolized, and eliminated unchanged through the kidneys. Clinical trials showed that pregabalin is an effective and safe analgesic, antiepileptic, and anxiolytic medicine [5–7].

During this period of two weeks, codeine and fentanyl were gradually discontinued. No side-effects were observed after the initiation of pregabalin, while no opioid withdrawal symptoms were reported. During pregabalin treatment, Mr. A.'s emotional state and painful symptoms improved progressively. On discharge, 6 weeks later the mean score in SF-McGill (I-a = 10, II = Mild, III = 1) (quality descriptors and intensity) was before the administration of pregabalin 26 and reduced to 11. The score in BDI reduced to 14. The patient reported on his last assessment that he could now enjoy his daily activities, be more active, and be less fearful of his pain symptoms that now were milder.

## 3. Discussion

To the best of our knowledge, this is the first case report of administration of pregabalin for the treatment of AS pain symptoms. Pregabalin, like gabapentin, was shown to be effective in several models of neuropathic pain (incisional injury, inflammatory injury, and formalin-induced injury) [5–7]. Chronic pathological pain is sustained by mechanisms of peripheral and central sensitization which are increasingly investigated at the molecular and cellular levels. The molecular mechanisms of sensitization that occur in peripheral nociceptors and the dorsal horns of the spinal cord are putative targets for context-dependent drugs, that is, drugs that are able to discriminate between “normal” and “pathological” pain transmission. Among these, pregabalin binds to the a_2_ *δ* subunit of voltage-sensitive Ca^2+^ channels, which sustain the enhanced release of pain transmitters at the synapses between primary afferent fibres and second order sensory neurons [7]. Although other anticonvulsants have been reported as helpful to patients with inflammatory conditions, pregabalin is particularly potent and effective as an analgesic and is characterized by linear kinetics across a wide range of doses. Similar to gabapentin, pregabalin is neither metabolized by cytochrome P450 (CYP) enzymes nor an inducer of drug-metabolizing enzymes. These features make pregabalin particularly suitable in terms of its lack of interaction with other drugs currently used for the treatment of chronic pain or associated psychiatric disorders (e.g., anxiety, depression) [7]. The antidepressive effect of pregabalin has been found to be associated with symptom relief in patients with generalized anxiety disorder [10]. Although Stein et al. in 2008 do report an antidepressive effect of pregabalin, one could postulate that the alleviation of the pain in our patient might be associated with the antidepressive effect of pregabalin, but this would be supported by the remarked improvement in his BDI scores. 

Opioids are widely used for their analgesic effects in several conditions associated with moderate to severe pain. Their use, however, can be limited by the dependence liability of this class of drugs, which is due in no small part to their reinforcing properties [11]. The production of the dependent state is coincident with the development of tolerance with chronic treatment and is associated with a physical withdrawal syndrome on abrupt cessation. Opioid tolerance develops because the brain cells that have opioid receptors on them gradually become less responsive to the opioid stimulation. For example, more opioid is needed to stimulate the ventral tegmental area brain cells of the mesolimbic reward system to release the same amount of dopamine in the nucleus accumbens [12]. These limitations explain why opioids are considered as drugs of a second choice according to the guidelines of the European Federation of Neurological Societies (EFNS) [13]. Therefore, it is of great importance to develop pharmacological treatments for pain for which there is little or no dependence liability. 

Opioids have in common the property of increasing dopamine levels within areas of the mesolimbic system. Opioid-induced increases in dopaminergic activity in mesolimbic regions are thought to enhance mood and motivation which in turn induces reinforcement and eventual dependence characterized by tolerance and a withdrawal syndrome following elimination of the drug from the plasma [11]. Pregabalin and gabapentin have no intrinsic rewarding properties; moreover, there are reports that opioid-induced reinforcement was prevented by blocking the increase in dopamine with either gabapentin or pregabalin pretreatment [14]. It is of notice that pregabalin has shown promising results in the treatment of alcohol dependence and in the discontinuation of long-term benzodiazepine use [15, 16], while gabapentin might be of use in the management of opioid withdrawal. The gabapentin daily dosages that have been reported as effective are 1800 mg [17]. The pregabalin dosages that have been administered successfully to patients of the existing studies [7, 10, 14, 15] have varied between 150 and 450 mg daily, as in the present case report, the dosage has been 300 mg.

Although anecdotal and requiring replication in controlled trials, the observations of the present report provide supportive preliminary evidence that pregabalin is potentially useful in the management of pain and comorbid psychiatric symptomatology in patients with complex medical problems such as AS and also in the treatment of withdrawal symptoms after the discontinuation of opioids.
